# The Effect of Molten Salt Composition on Carbon Structure: Preparation of High Value-Added Nano-Carbon Materials by Electrolysis of Carbon Dioxide

**DOI:** 10.3390/nano15010053

**Published:** 2024-12-31

**Authors:** Yi Cheng, Liangxing Li, Lirong Xue, Jiahang Wu, Jingsong Wang, Xilin Huang, Chunfa Liao

**Affiliations:** 1 School of Energy and Mechanical Engineering, Jiangxi University of Science and Technology, Nanchang 330013, China; 6720221118@mail.jxust.edu.cn (Y.C.); 6720221117@mail.jxust.edu.cn (L.X.); 6720231559@mail.jxust.edu.cn (J.W.); 6120231533@mail.jxust.edu.cn (J.W.); 2 Institute of Metallurgical and Chemical Engineering, Jiangxi University of Science and Technology, Ganzhou 341000, China; liaochfa@163.com

**Keywords:** carbon dioxide, molten salt electrolysis, carbon materials, chloride molten salt

## Abstract

The electrochemical conversion of CO_2_ into high value-added carbon materials by molten salt electrolysis offers a promising solution for reducing carbon dioxide emissions. This study focuses on investigating the influence of molten salt composition on the structure of CO_2_ direct electroreduction carbon products in chloride molten salt systems. Using CaO as a CO_2_ absorber, the adsorption principle of CO_2_ in LiCl-CaCl_2_, LiCl-CaCl_2_-NaCl and LiCl-CaCl_2_-KCl molten salts was discussed, and the reasons for the different morphologies and structures of carbon products were analyzed, and it was found that the electrolytic efficiency of the whole process exceeded 85%. Furthermore, cathode products are analyzed through Scanning Electron Microscope (SEM), X-Ray Diffractometer (XRD), Thermal Gravimetric Analyzer (TGA), Raman Spectra and Fourier Transform Infrared (FTIR) techniques with a focus on the content and morphology of carbon elements. It was observed that the carbon content in the carbon powder produced by molten salt electrochemical method exceeded 99%, with most carbon products obtained from electrolysis in the Li-Ca chloride molten salt system being in the form of carbon nanotubes. In contrast, the Li-Ca-K chloride system yielded carbon nanospheres, while a mixture was found in the Li-Ca-Na chloride system. Therefore, experimental results demonstrate that altering the composition of the system allows for obtaining the desired product size and morphology. This research presents a pathway to convert atmospheric CO_2_ into high value-added carbon products.

## 1. Introduction

In recent years, with the development and progress of science and technology, there has been an increasing demand for high-performance materials. Carbon nanotubes (CNTs) have garnered significant attention due to their excellent performance as electrode material for Li-ion batteries, exhibiting higher lithium capacity than graphite and good cycle performance. The hollow and seamless tubular structure of carbon nanotubes, composed of single or multilayer curling flakes of graphite, including multi-walled and single-walled varieties, has sparked widespread scientific interest. In terms of the advantages of CNT and MWCNT, single-walled nanotubes and multi-walled nanotubes, their graphene cylinders are arranged differently. Single-walled carbon nanotubes have only one graphene cylinder, while MWNTs have many layers. This makes their application fields different, comparing the characteristics of cathodes containing various types of commercially available carbon nanotubes. Cathode materials containing SWCNTs show the best electrochemical properties. In addition to electrical conductivity, carbon materials also have good thermal conductivity. Surakasi et al. dispersed MWCNTs in an aqueous fluid of TiO_2_, which increased the thermal conductivity of TiO_2_ by 10–18%, and further expand research on various forms of carbon materials with unique functions such as carbon nanospheres and graphene [1]. Carbon black has important application value in environmental treatment. Due to its excellent adsorption and renewable properties, carbon black is widely used in wastewater treatment, gas adsorption and solid waste treatment. Carbon nanospheres with the same high specific area and adsorption capacity can also be used as water purification agents, and carbon black is mainly used as a reinforcing agent and filler for rubber, especially in tire manufacturing. Carbon nanotubes can be incorporated into tires, membranes, coatings, batteries and many other applications. In most large-scale production applications, and they perform better [2,3]. Methods for preparing carbon materials include laser and arc evaporation of graphite, chemical vapor deposition, and molten salt electrolysis [4,5]. These methods aim to obtain carbon atoms or clusters from carbon-containing substances which then interact with other substances to grow carbon materials with distinctive physical and chemical properties [6].

The utilization of carbon dioxide as a C_1_ resource has gained increasing attention due to its significance as the main greenhouse gas [7]. Research in this area aims to reduce carbon dioxide emissions and lower its atmospheric concentration by converting it into high value-added carbon materials. However, the thermodynamic stability of CO_2_ makes it challenging to disintegrate. Current methods for addressing this challenge include photochemical and biological carbon sequestration [8]. While both approaches have shown promising capabilities for degrading CO_2_, their practical application is hindered by complex experimental processes and high costs.

With the advancement of molten salt electrochemistry, high-temperature molten salt is utilized to capture and convert CO_2_ into high value-added carbon materials through electrochemistry, or alkanes or syngas produced by electrolysis with other compounds [9,10,11,12]. This approach offers a sustainable and environmentally friendly method for converting CO_2_ into valuable materials. During the chemical reaction process, electrons serve as powerful redox reagents to effectively reduce CO_2_, resulting in the creation of various carbon products with unique physical and chemical properties including energy materials, carbon nanotubes, carbon nanofibers, hollow carbon spheres, and carbon nanoparticles [13,14,15]. Furthermore, this method can be employed to create carbon film coatings on electrode surfaces [16,17]. It is important to note that due to temperature influences during the process, the resulting product morphology mainly consists of solid carbon and CO. When the temperature is lower than 750 °C, the main reduction product is carbon powder, accompanied by a small amount of CO gas generation; the molar ratio of solid carbon to gas is 20:1. With the gradual increase in temperature, the CO content increases, and almost only CO gas is generated when it exceeds 950 °C. Nanoscale amorphous carbon and rod-shaped graphite crystals are the primary constituents of solid carbon. These carbon materials exhibit excellent conductivity and adsorption properties, which make them suitable for use as electrode materials in energy equipment [9,18,19] or as active adsorbents for capturing harmful substances [20]. The common reduction method is indirect reduction in carbonate systems [21,22]; the principle is that the CO_3_^2−^ is converted into C and O^2−^ on the surface of the cathode by the action of electrons. Part of O^2−^ is free to the anode to be converted into O_2_ and the other part is free to the surface of molten salt and combines with CO_2_ to CO_3_^2−^. Additionally, there is a theory of direct reduction in which CO_2_ is captured in a chloride salt system and then reduced to solid carbon at the cathode. Compared with indirect reduction, direct reduction has a lower temperature. According to Novoselova’s [6] analysis of configurations in Li^+^ and Cl^−^ media, it was observed that chloride ions remain in their original positions, while lithium ions move downward to the edge of the graphene plane and occupy positions outside of that plane. The interaction between graphene fragments loaded with Li^+^ and Cl^−^ ions results in a natural distortion of the graphene plane, forming cylindrical structures such as nanotubes with open ends. Therefore, the ionic medium of alkali metal chloride melt is advantageous for facilitating the rotation of flat graphene planes within cylinders and the formation of CNT nuclei.

This article focuses on the direct reduction of CO_2_ in various chlorinated systems at a temperature of 750 °C. It also investigates the reaction mechanism of electrodes and the composition and structure of cathode products. The study aims to explore the impact of various molten salt systems on the morphology of carbon products and to develop methods for the controlled conversion of CO_2_ into high value-added carbon materials.

## 2. Experimental Process

### 2.1. Experimental Electrolyte System

**Figure 1:**
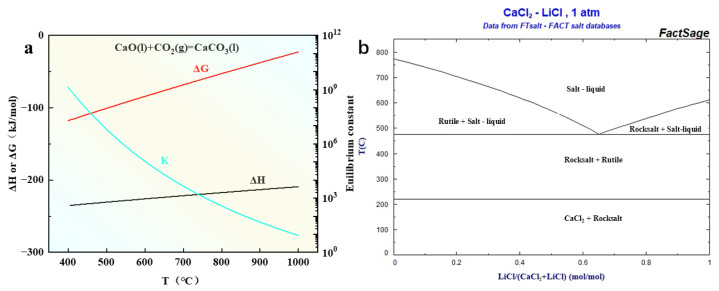
(**a**) The thermodynamic data of the reaction between CaO and CO_2_ calculated using HSC software. (**b**) Binary phase diagram of CaCl_2_-LiCl from Factsage.

The presence of Li^+^ is crucial for the deposition of carbon in molten salts, because the standard potential for the electrochemical conversion of Li^+^ to metallic lithium is more negative than that for the conversion of CO_2_ to elemental carbon. The standard potentials for the electrochemical conversion of Na^+^ and K^+^ to corresponding metals are close to or more positive than the standard potentials for the conversion of CO_2_ to elemental carbon, respectively, indicating that the reduction of Na^+^ and K^+^ will compete with the carbon deposition, which is detrimental to the carbon reduction reaction. Therefore, it is more feasible to obtain carbon by electrolysis in a molten salt electrolyte system containing Li^+^ [23]. Novoselova [6] found that the solubility of CO_2_ in chloride salt systems is limited, with a solubility of 0.48 × 10^−6^ in NaCl and 0.57 × 10^−6^ in KCl at 750 °C. The solubility of CO_2_ in alkaline earth metal compounds exceeds that in alkali metal chloride salts. During the experiment, the adsorption of CO_2_ was carefully considered, leading to a preference for electrolytes with superior CO_2_ absorption performance, as shown in Figure 1a. Thermodynamic data were calculated using HSC 5.0 software, revealing that CaO, similar to Li_2_O, exhibits significant capacity for CO_2_ absorption [24]. Furthermore, calcium-based molten salts possess a wide electrochemical window in the reaction process, which is conducive to electrolytic reaction and impurity pretreatment. In the chloride molten salt system, carbon dioxide absorption primarily depends on O^2−^ generated by CaO dissolving into molten salts and undergoing reactions described by Equations (1) and (2) within the molten salt medium. From an economic standpoint, choosing CaO as a CO_2_ absorber is more advantageous (if not specified later, CaO is added to the molten salt system by default, and the added mass is 2 wt% CaCl_2_).

CaO = Ca^2+^ + O^2−^ (1)

O^2−^ + CO_2_ = CO_3_^2−^ (2)

### 2.2. Experimental Operation

In the experiment, anhydrous LiCl and CaCl_2_ of analytical purity were combined in a molar ratio of 0.65:0.35 to form a Li-Ca chloride molten salt electrolyte, as illustrated in Figure 1b. The anhydrous electrolyte was meticulously mixed and then subjected to 12 h drying process at 200 °C before being transferred into a nickel crucible, which was subsequently placed in a stainless-steel crucible. Under standard atmospheric pressure, CO_2_ was continuously introduced into the crucible at a flow rate of 1 LPM, and the temperature was gradually increased to 300 °C and maintained for 2 h to ensure complete removal of moisture. Subsequently, the temperature was raised at a controlled rate of 2 °C/min until reaching 750 °C. Electrolysis took place between the Pt anode and the Ni cathode. Initially, a current density of 10 mA/cm^2^ was applied for the pre-electrolysis process before expanding to 200 mA/cm^2^ for rapid carbon deposition. After electrolysis for 2 h, disconnect the power supply, stop heating, and stop ventilation when it cools to room temperature. The electrolysis process for LiCl-CaCl_2_-KCl (0.65:0.35:0.25) and LiCl-CaCl_2_-NaCl (0.65:0.35:0.25) systems can be carried out in a similar manner. Throughout the entire experiment, the energy needed for heating and electrolysis can be substituted with solar energy [25,26]. This approach ensures environmental sustainability while minimizing harm to the ecosystem. See Supplementary Information Figure S1 for the electrolysis equipment.

### 2.3. Experimental Methods

Characterization of carbon materials. The electrochemical analysis of the entire reaction was performed using Princeton 4000A, the resulting carbon products were characterized using X-ray diffraction (XRD, Rigaku SmartLab SE, Akishima, Japan) at a scanning rate of 2°/min at 40 kV and 150 mA Cu-K α radiation, and Raman imaging was performed with a TEM single-frequency laser (λ = 532 nm, laser power = 40 mW, Witec GmbH, Ulm, Germany). Microstructure and microchemical analysis of deposited carbon were observed by scanning electron microscopy (SEM, ZEISS MajiniSEM 300, Jena, Germany) and energy spectrometer (EDS, Octane Elect Super + Hikari Plus). In addition, Fourier infrared spectroscopy (FTIR, Thermo Fisher Science Nicolet iS20, Waltham, MA, USA) and thermogravimetric analysis (TG, Netzsch STA 449, Selb, Germany) were used to analyze the functional groups that carbon products may contain.

## 3. Result Analysis

### 3.1. Conversion of Carbon Dioxide

**Figure 2:**
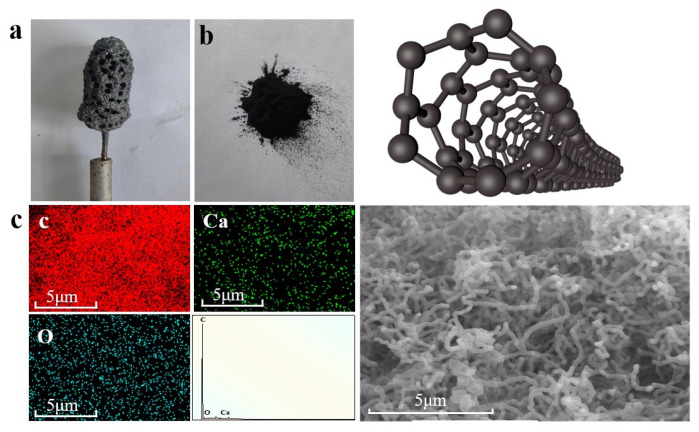
(**a**) The products attached to the Ni electrode after electrolysis. (**b**) The product subsequent to acid leaching and purification treatment (on the right is a schematic diagram of the structure of CNTs using C4D). (**c**) SEM map and element distribution obtained after purification.

The product obtained in the Li-Ca chlorination system, as shown in Figure 2a, obtains a thick layer of carbon product on the cathode and white particles appear on the surface of the product surface. This is because CO_3_^2−^ decomposes into C and O_2_ on the surface of the Ni cathode under the action of electrons, and the reaction of Equations (3)–(6) occurs. The white particles are due to the fact that when the cathode is lifted, some molten salts may adhere to the product, so acid leaching treatment is a necessary step. Subsequently, hydrochloric acid is added to disperse the product thoroughly with ultrasound and soaked for 12 h. The resulting mixture is rapidly filtered using a suction filter while continuously rinsing with deionized water during the filtration process. After adequate rinsing, it is dried at 200 °C in a drying oven. Figure 2b shows the product after undergoing acid leaching treatment. Upon detection using EDS (Energy Dispersive Spectroscopy), it was found that carbon content exceeded 90%, with a small amount of oxygen and calcium present. Through multiple repeated experiments, it was observed that the ratio of carbon to calcium consistently approached similar values. Abbasoo [27] speculated that Ca alkali metal may have been inserted into the graphite interlayer, while the oxygen element might exist as an oxygen-containing functional group. In order to obtain higher-purity carbon products, in the acid leaching treatment, stirring at 60 °C for 12 h, and prolonging the time and number of suction filtration, followed by drying overnight, the samples were placed in a tube furnace, heated to 800 °C at 5 °C/min under an atmosphere of H_2_/Ar (10%), and kept warm for 2 h. The content of Ca and C in the product was reduced in the above manner. It was proved that after the above treatment, the carbon content reached 99.83%. The morphology image of the carbon material after treatment and the Figure S2 with additional information on the element content.

### 3.2. Electrochemical Analysis

As shown in Figure 2, we have successfully achieved the conversion of CO_2_ into elemental carbon. To investigate the conversion mechanism of CO_2_, two sets of experiments were conducted. One involved a blank system without the addition of CaO to the molten salt, while the other involved an experimental system with CaO added as a trapping agent. To ensure a meaningful comparison and minimize potential CO_2_ interference, the blank system was operated at 750 °C in an Ar atmosphere, while the experimental system was operated at 750 °C in a CO_2_ atmosphere.

**Figure 3:**
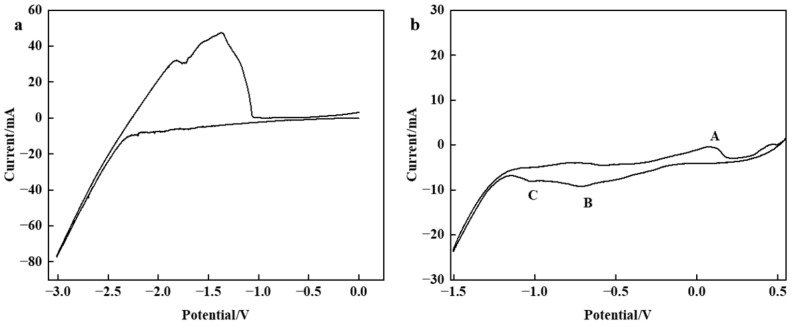
Cyclic voltammetry curve recorded at 750 °C at scanning speed of 10 mV/s using Ni wire as working electrode, carbon rod as counter electrode, Pt wire as reference electrode, (**a**) LiCl-CaCl_2_ system in Ar atmosphere, (**b**) LiCl-CaCl_2_ system in CO_2_ atmosphere.

The process of carbon deposition was studied by cyclic voltammetry, in which a nickel wire was employed as the working electrode, a glassy carbon rod served as the counter electrode, and a platinum wire functioned as the reference electrode. As shown in Figure 3a, during the negative scanning at 10 mV/s, no reduction peak was observed at 0–1.5 V (vs. Pt), indicating that no reaction occurred in this range. And the reduction potential of CO_2_ is also in this range, so the range of subsequent tests is selected within this range. The result of the cyclic voltammetry test for the blank system indicates that molten salt electrolyte and electrodes remain stable within the range of −1.5 to 0 V. The results of the cyclic voltammetry at the same sweep speed for the experimental system are shown in Figure 3b. Two reduction peaks, B and C, are observed at potentials −0.6 V (vs. Pt) and −1.1 V (vs. Pt), This may mean that the reduction of CO_3_^2−^ is divided into two steps. Reaction (4) occurs first. The intermediate CO_2_^2−^ generated is extremely unstable, releasing CO, and then reacting CO at the cathode to obtain C, resulting in reactions (5) and (6). The oxidation peak A observed during forward scanning corresponds to CO_3_^2−^ oxidation. These findings indicate that the following reactions may occur throughout the reaction process.

Oxidation stage:

2CO_3_^2−^ → 2CO_2_ + O_2_ + 4e^−^ (3)

Reduction stage:

CO_2_ + 2e^−^ → CO_2_^2−^ (4)

CO_2_^2−^ → CO + O^2−^ (5)

CO + 2e^−^ → C + O^2−^ (6)

### 3.3. Products Obtained from Electrolysis of CO_2_ in LiCl-CaCl_2_ Molten Salt

**Figure 4:**
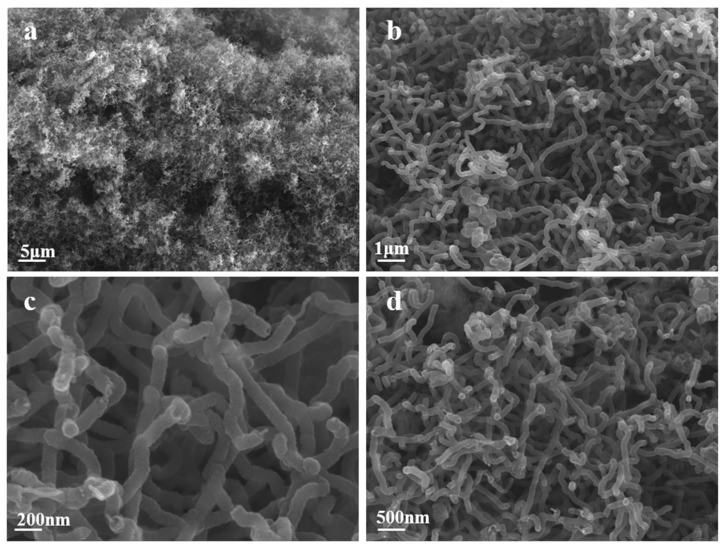
SEM image of the product obtained from electrolysis of CO_2_ in LiCl-CaCl_2_ molten salt at 750 °C, the magnifications are (**a**) 2000 times; (**b**) 10,000 times; (**c**) 50,000 times; (**d**) 20,000 times.

In this experiment, a nickel wire with a diameter of 1.5 mm was utilized as the working electrode, while a glassy carbon rod with a diameter of 1.5 mm was employed for electrolysis at 750 °C. Electrolysis was performed at a current density of 10 mA/cm^2^ for half an hour, followed by an increase in the electrolysis to 200 mA/cm^2^ for one hour to facilitate rapid carbon deposition. The Coulomb efficiency of the electrolysis process is determined by calculating the actual weight of carbon deposited on the cathode and the electricity consumed during the electrolysis process. It was noted that in the Li-Ca chloride system, the Coulomb efficiency exceeded 85%. The morphology of the product obtained through constant current electrolysis in the Li-Ca chloride system at 750 °C is shown in Figure 4. Numerous large-area entangled CNTs are observed in the chloride molten salt of Li-Ca, as shown in Figure 4a,b, which are much more abundant than those found in the chloride salt systems of Li-Ca-Na and Li-Ca-K. As indicated in Figure 4c, the diameter thickness of CNT in Li-Ca chloride salt ranges from approximately 50 to 100 nm, which is also finer than that in Li-Ca-Na. In comparison to the carbonate used by Li [28] while maintaining the cation phase, the product size obtained in the chloride system is similar, but with better uniformity of size than that in the carbonate system. In Figure 4d, a SEM image magnified 20,000 times reveals the clear morphology of CNTs. The smooth and defect-free CNTs can significantly enhance electron transport within the tube, thereby endowing them with high conductivity and making them suitable for use as electronic materials in lithium-ion batteries. In addition to the influence of the electrolyte, electrode composition also affects the product. In pure Li_2_CO_3_ molten salt experiments, Douglas et al. [29] observed using nickel plated with Al_2_O_3_ as the anode and waste brass and stainless steel as cathodes resulted in finer and more concentrated diameters of electrolyzed CNTs. This demonstrates that electrode material has a certain impact on product morphology. Licht [30] observed during their experiments that capacity gradually increases during the battery cycling process due to defective CNTs’ modification induced by defects stored during cycling process procedures. Due to their outstanding conductivity and modification properties, CNTs are widely utilized in the field of batteries. Additionally, they also have applications in biotechnology, where they are used to improve the environment and aid in the development of artificial tissues [31].

### 3.4. Products Obtained from Electrolysis of CO_2_ in LiCl-CaCl_2_-NaCl and LiCl-CaCl_2_-KCl Molten Salt Systems

**Figure 5:**
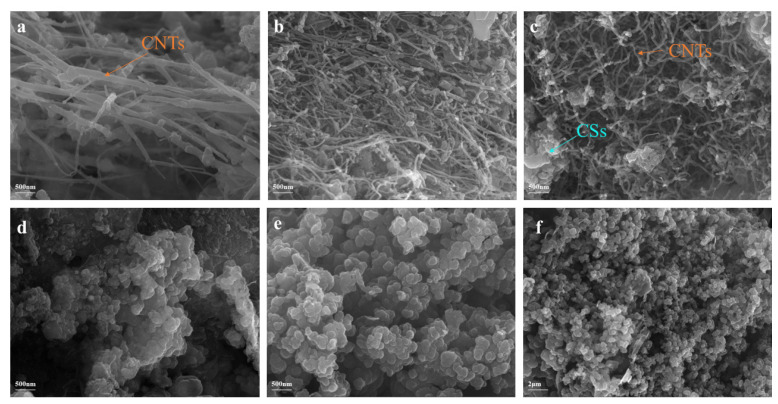
At 750 °C, the system was electrolyzed at a constant current of 200 mA/cm^2^: (**a**) SEM images of the products obtained from electrolysis of CO_2_ in LiCl-NaCl molten salt, (**b**,**c**) SEM images of the products obtained from electrolysis of CO_2_ in LiCl-CaCl_2_-NaCl molten salt, (**d**) SEM images of the products obtained from electrolysis of CO_2_ in LiCl-KCl molten salt, (**e**,**f**) SEM images of the products obtained from electrolysis of CO_2_ in LiCl-CaCl_2_-KCl molten salt.

Figure 5 illustrates the morphology of the products produced by electrolysis using a glassy carbon rod anode and Ni cathode in LiCl-CaCl_2_-NaCl and LiCl-CaCl_2_-KCl at a temperature of 750 °C. In order to control the influence of other variables, the electrode size and current density selected are consistent with those in the Li-Ca chloride molten salt experiment. In the Li-Ca-Na chloride system, as shown in Figure 5b,c, the morphology of the products is a combination of carbon nanotubes (CNTs) and carbon spheres (CSs). Meanwhile, Figure 5e,f show that in the Li-Ca-K system, CSs are the predominant morphology with a small quantity of CNTs mixed in. The principle of this method is that a part of CO_2_^2−^ is converted into carbon material, and the remaining CO_2_^2−^ enters the carbon material and is reduced to CO gas. As the gas increases, the carbon material continues to expand, eventually forming the structure of carbon nanospheres. It is noted that compared to LiCl-CaCl_2_-NaCl, the size of CSs is more uniform in the LiCl-CaCl_2_-KCl system. A comparison between Figure 4 and Figure 5 reveals that the arrangement of CNTs in both systems involving Na^+^ or K^+^ is disorderly, with their quantity and smoothness being inferior to those observed in solely Ca^2+^. This suggests that Na^+^ and K^+^ have an inhibitory effect on CNT growth while promoting CS formation due to their reduction potential requirement related to corresponding alkali metals. Due to its unique electronic properties, adsorption capabilities, and most importantly, its customized structure, CS has the potential to be applied in different technological fields, such as lithium batteries, catalyst carriers for drug delivery, encapsulation of active transition metals, and adsorbents. The synthesis of CNTs and CSs can be controlled by adjusting the content of KCl and NaCl as necessary. In comparison to CNTs in the Li-Na chloride system, CNTs in the Li-Ca-Na chloride system exhibit a slightly smaller diameter compared to Figure 5a,c, There is a similar situation in the synthesis of carbon nanospheres, where the product in the system containing CaCl_2_ is more uniform and smaller in size. Compare Figure 5d,e. In the past, many studies have tried to controllably prepare carbon products with different morphologies, such as Yu et al. [32] trying to synthesize carbon nanotubes or carbon nanospheres by controlling different conditions. However, the products electrolyzed in carbonates are often not pure enough, and are mixed with various forms, even amorphous carbon. In this paper, electrolysis in chlorinated salts can obtain higher-purity carbon nanotubes or carbon nanospheres, and the size is smaller and more uniform.

**Figure 6:**
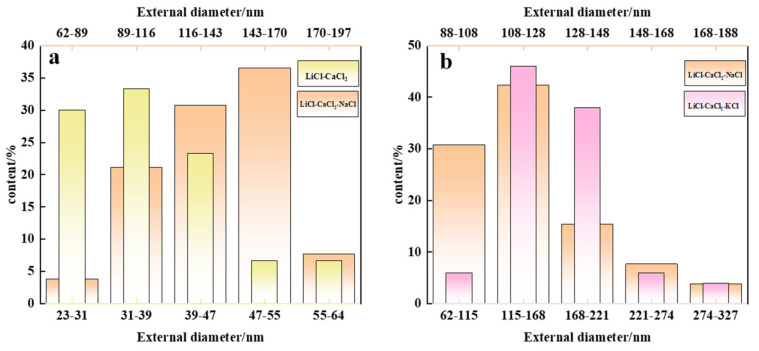
The outer diameter distribution of products obtained at a current of 200 mA/cm^2^ and 750 °C under different electrolyte systems: (**a**) CNTs in Li-CaCl_2_ and CNTs in Li-CaCl_2_-NaCl, (**b**) CSs in Li-CaCl_2_-KCl and CSs in Li-CaCl_2_-NaCl.

We utilized Nano Measure 1.2 software to compute the distribution of outer diameters of carbon products acquired from various electrolyte systems, as depicted in Figure 6. It is evident that the size of the product varies with different electrolytes. Specifically, for CNTs, the size is concentrated in the range of 31.2–55 nm in the LiCl-CaCl_2_ system, with an average size of 45 nm, while in the LiCl-CaCl_2_-NaCl system, it is concentrated in the range of 62–143 nm with an average size of 111 nm, as shown in the Figure 6a. Additionally, a comparison of CSs is presented in Figure 6b. The average outer diameter in LiCl-CaCl_2_-KCl is also smaller than that in LiCl-CaCl_2_-NaCl.

### 3.5. XRD Analysis of Products

**Figure 7:**
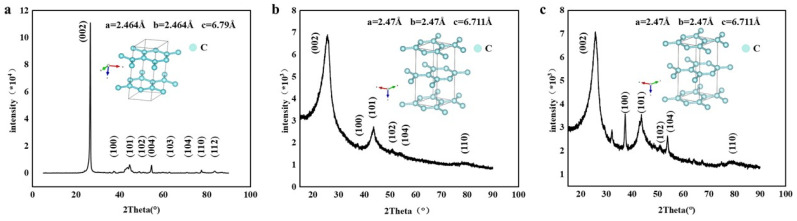
The XRD diagram of the product obtained by (**a**) LiCl-CaCl_2_, (**b**) LiCl-CaCl_2_-NaCl, (**c**) LiCl-CaCl_2_-KCl and its corresponding unit cell type and parameters in 750 °C at a current of 200 mA/cm^2^.

In order to further elucidate the composition of the product, X-ray diffractometry was used to analyze the crystal structure. The X-ray diffraction patterns of the carbon products prepared in Li-Ca, Li-Ca-Na, and Li-Ca-K chloride systems are shown in Figure 7a–c, respectively. These patterns were compared with the standard card of graphite, revealing a distinct carbon characteristic peak at 26°. The corresponding (002), (100), and (101) carbon lattice planes reflect differently in relative intensity and width. The reflection width of Li-Ca chloride systems is much narrower than that of Li-Ca-Na and Li-Ca-K chloride systems, indicating that the crystal in Li-Ca is larger than that of Li-Ca-Na and Li-Ca-K [33]. It is notable that the half peak width is larger in the chloride systems of Li-Ca-K and Li-Ca-Na, as shown in Figure 7b,c, indicating that the grain size of the product is relatively fine and disordered, resulting in a lower level of graphitization. Consistent with previous experiments, it was observed that KCl and NaCl may interfere with the graphitization process, as evidenced by a lower XRD peak for products prepared in Li-Ca-K compared to those from Li-Ca-Na. This suggests a lower crystallinity for products from the Li-Ca-K system due to uncontrollable multi-dimensional growth orientation induced by K^+^. Furthermore, this test also confirmed a higher proportion of CNTs in products from the Li-Ca-Na chloride system compared to those from Li-Ca-K. Upon removing interference from KCl and NaCl during experiments, there was an observed increase in CNT proportion within the product. The peak of the product prepared in the Li-Ca system is very sharp at 26°, which is significantly higher than the XRD peak of the product in the Li-Ca-K and Li-Ca-Na systems, as shown in Figure 7a. This indicates that the crystallinity and graphitization level of the product are high, leading to a high fraction of CNTs grown. In addition, the (100) peak represents the arrangement of the aromatic ring. In high-quality graphitized materials, the (100) peak intensity is weaker, while in low-quality carbon materials, it may show stronger strength. The (101) peak around 43° represents the layer spacing of the graphite material, and its strength will gradually decrease as the layer spacing increases. As shown in Figure 7, the obtained carbon material in the Li-Ca-K system has a relatively low degree of graphitization and a higher number of layers.

### 3.6. Analysis of Products by TGA and FTIR

**Figure 8:**
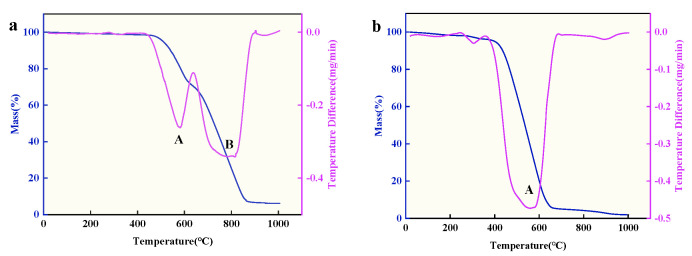
The TG-DTG thermograms of carbon materials, recorded at a heating rate of 10 °C/min at a low air velocity of 100 milliliters per minute: (**a**) CNTs, (**b**) CSs.

First, the product is to be dried in an incubator at 100 °C for 12 h. Subsequently, the stability of the CNT prepared in Li-Ca chloride salt and CS in the Li-Ca-K system is to be tested using TGA at a heating rate of 10 °C/min in an atmospheric environment. The results are presented in Figure 8. DTG refers to the rate of weight loss per unit of time, with various peaks on the DTG curve corresponding to each weight change stage on the TG curve. The peaks at points A and B represent distinct weight change stages near these points. In comparison with the mass loss of CS starting at 400 °C, it is observed that CNT still exhibits good stability at temperatures exceeding 450 °C. The TGA diagram in the Li-Ca chloride system has two weight change points. Due to the previous EDS data, it can be guessed that CaCO_3_ is generated in this process, which delays the rate of product quality decline. Upon completion of heating, a little residue remains in the corundum crucible in the Li-Ca chloride system. As mentioned earlier, this indicates that the carbon material is mainly composed of carbon with oxygen-containing functional groups and a little calcium.

**Figure 9:**
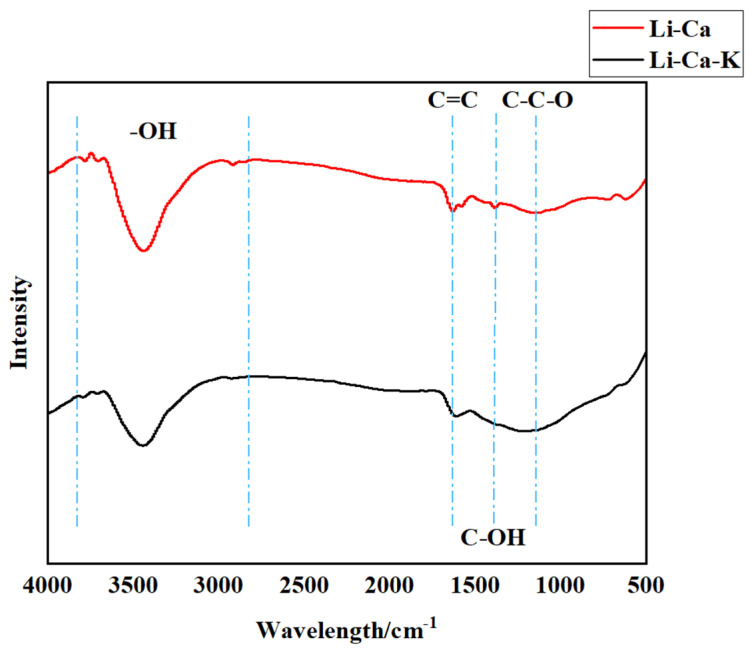
FTIR spectra of CNT and CS.

The CSs prepared in the Li-Ca-K chloride system and CNTs prepared in the Li-Ca chloride system were analyzed using infrared spectroscopy. The results of the analysis are shown in Figure 9. The peak observed at 1110 cm^−1^ corresponds to the stretching vibration of the C-C-O functional group, indicating the presence of oxygen in the EDS product mentioned earlier and confirming our speculation about the existence of an oxygen-containing functional group. Additionally, a peak at around 1600 cm^−1^ corresponding to C = C, as an unsaturated hydrocarbon, could be attributed to the excellent conductivity of CNTs and CSs. Between 2700 cm^−1^ and 3700 cm^−1^, there is a large peak and many small peaks, which are related to the vibration of C-OH. The H here may come from long-term immersion in water, so a small part of the H in the water combines with the oxygen-containing functional group of the carbon material to form -OH. In addition, there is also a vibration peak at 1383 cm^−1^, representing the C-OH functional group [34].

### 3.7. Raman Analysis of the Product

**Figure 10:**
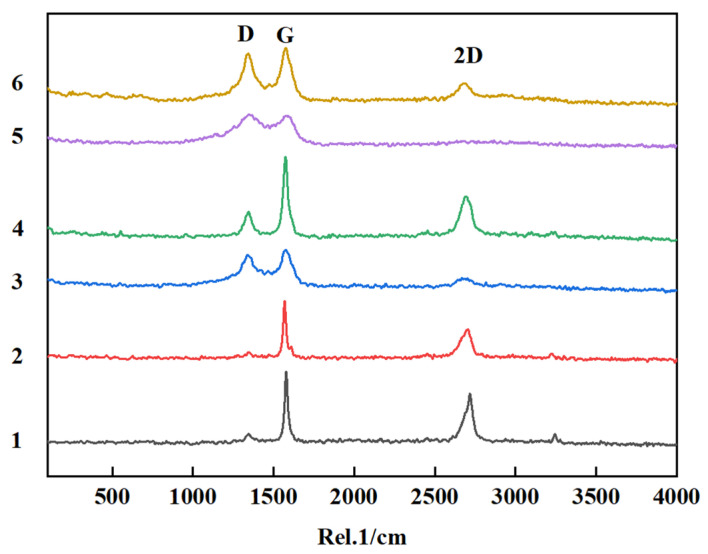
Raman characterization of products in different systems under He-Ne laser wavelength of 532 nm. Raman spectra of electrolytic products in different systems; 1 and 2 are in LiCl-CaCl_2_ system, 3 and 4 are in LiCl-CaCl_2_-NaCl system, 5 and 6 are in LiCl-CaCl_2_-KCl.

Evaluate the degree of graphitization of synthesized CSs and CNTs using Raman spectroscopy. Raman imaging was performed with a confocal Raman microscope equipped with a TEM single-frequency laser (λ = 532 nm, laser power = 40 mW), as shown in Figure 10. The most striking Raman bands of carbon materials and their composites are the D-band (1300~1400 cm^−1^), G-band (1580 cm^−1^), 2D-band (also known as G ‘band, 2600~ 2700 cm^−1^). These Raman bands outperform many other Raman bands in strength, which allows us to maintain a certain degree of accuracy in the analysis process, but in addition to these commonly used bands, there are other Raman bands with lower peaks that also contain rich information, such as the C-band (40 cm^−1^) and the D-band (1620 cm^−1^) [35]. Both Li-Ca and Li-Ca-K displayed two broad and high peaks, which are characteristic of carbon materials. Specifically, the intensity of the D peak indicates the proportion of defects, structural distortions, and amorphous components in the sample. This can provide information on the material lattice structure, impurities, and content of amorphous components in the sample. In addition, it is important to note that there is a weak peak associated with the D peak, commonly known as the T peak. This peak indicates that some carbon atoms in the product are in the sp^3^ hybridized state. The G peak, which occurs between 1580 and 1595 cm^−1^, is caused by the in-plane vibration of the C-C bond. The intensity of the G peak reflects both the number of layers and lattice integrity of the material. In single-layer structures such as graphene, there is a significant enhancement phenomenon observed. The shape and position of peaks are related to various factors such as bonding mode, lattice vibration, and optical properties of sp^2^ carbon atoms in the material. These characteristics can be used to analyze and characterize properties such as material quality, lattice, and band structure within the sample. Furthermore, Raman spectra obtained from several points on a single sample are nearly identical, indicating uniformity within the prepared sample. Additionally, the ratio of the intensities between D and G peaks (*I*_D_/*I*_G_) can provide insight into crystallinity; smaller values suggest higher crystallinity, while larger values indicate lower crystallinity and more disorder within the product. For instance, the *I*_D_/*I*_G_ values for Li-Ca system carbon nanotubes fall within a range from 0.7 to 0.9, which aligns with commercial carbon nanotube standards. Furthermore, the *I*_D_/*I*_G_ values for K-induced interference and CaO-induced interface modification lead to higher surface disorder advancing up until approximately 1.0.

The peak at around 2700 cm^−1^ is a characteristic feature of graphene, commonly referred to as the 2D peak. This particular peak is often utilized to determine the number of layers present in graphene, and the calculation method involved is denoted as *I*_2D_/*I*_G_. As the proportion decreases, it indicates that the graphene layer is becoming progressively thicker [35].

## 4. Conclusions

In this paper, we try to electroreduce CO_2_ in different chloride systems; the reaction mechanism and the morphology and phase composition of carbon products were analyzed by CV, SEM, XRD, etc. The results proved that it is a feasible scheme for electrochemical reduction of CO_2_ to carbon products in chloride systems, and the obtained carbon products have a smaller crystal size. The thermodynamic efficiency exceeded 85% during the electrolysis process. XRD and TGA analysis confirmed that most of the prepared products were carbon materials, which proved the feasibility and advantage of electrochemical preparation of carbon materials using molten salts, as shown in Table 1. SEM revealed that variations in electrolyte composition led to differences in the morphology of the synthesized carbon material. We observed that K^+^ and Na^+^ had a promoting effect on the growth of CSs, with K^+^ exerting a stronger inhibitory effect on the growth of CNTs. Thus, by reasonably controlling the composition of the electrolyte, we can tailor our synthesis process to produce carbon materials with diverse morphologies according to specific requirements.

As mentioned earlier, the use of renewable energy sources (such as solar energy) to prepare carbon dioxide into high value-added by-products holds great promise in industrial applications. The flue gas emitted by modern industry not only contains a variety of gases, but the emission temperature is also high, which makes the means of enriching carbon dioxide very expensive. High-temperature molten salt has a strong adsorption capacity for CO_2_, and the adsorption cost is also very low; in addition, high-temperature flue gas can reduce the energy required for heating after entering. Therefore, the use of molten salt electrolysis to directly electrolyze carbon dioxide to bypass the enrichment of carbon dioxide can not only greatly reduce the cost of capture and purification, but also alleviate the global climate crisis. 

## Figures and Tables

**Table 1:** Summary of product morphology and efficiency in different molten salt systems at 750 °C.

System	Cathode	Anode	Product Structure	Efficiency/%
LiCl-CaCl_2_	Ni	Glassy Carbon	CNT	88
LiCl-CaCl_2_-NaCl	Ni	Glassy Carbon	CNT, CS	82
LiCl-CaCl_2_-KCl	Ni	Glassy Carbon	CS	75

## Data Availability

The data that support the findings of this study are available from the corresponding author upon reasonable request.

## Supplementary Materials

The following supporting information can be downloaded at: https://www.mdpi.com/article/10.3390/nano15010053/s1, Figure S1: Schematic diagram of the heating and electrolysis equipment used in the experiment; Figure S2. SEM and EDS pictures of purified products, (a) The element content of the product after purification; (b) The EDS map of the purified product, with carbon on the left and oxygen on the right; (c) SEM image of the purified product.
