# Supplementary material for: The Effect of Molten Salt Composition on Carbon Structure: Preparation of High Value-Added Nano-Carbon Materials by Electrolysis of Carbon Dioxide
Source: Nanomaterials (Basel). 2024 Dec 31;15(1):53. doi: 10.3390/nano15010053 (PMC11723251; doi:10.3390/nano15010053)
Supplement: Supplementary file 1 [file nanomaterials-15-00053-s001.zip › nanomaterials-3309140-supplementary.pdf]

The Effect of Molten Salt Composition on Carbon Structure: Preparation of High Value-Added Nano-Carbon Materials by Electrolysis of Carbon Dioxide

S1. Experimental instrument

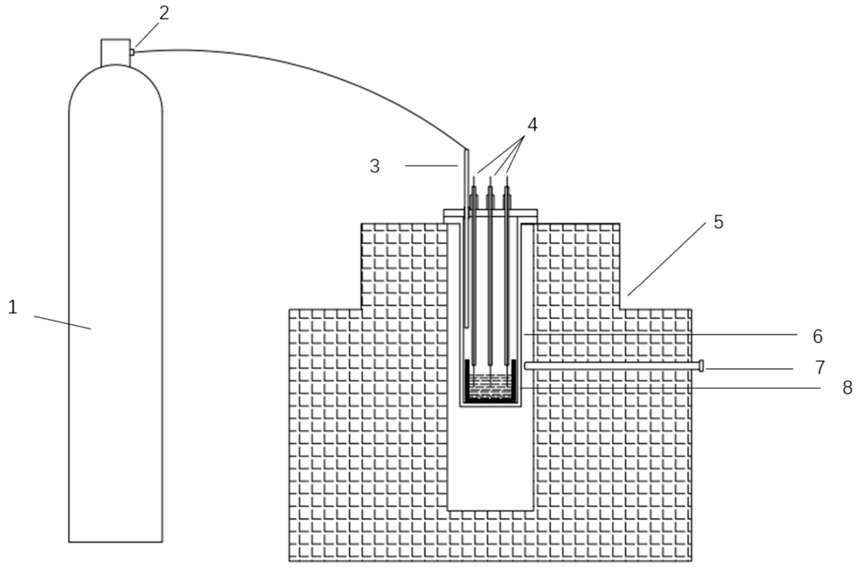

1: gas cylinder; 2: control valve; 3 vent tube; 4: electrode (working electrode, reference electrode, counter electrode are respectively Ni, Pt, graphite rod) 5: resistance furnace; 6: stainless steel crucible; 7 thermocouple; 8 graphite crucible.

Figure S1. Schematic diagram of the heating and electrolysis equipment used in the experiment.

S2. Purification of samples

In order to obtain higher purity carbon products, in the acid leaching treatment, stirring at 60 °C for 12 h, and prolonging the time and number of suction filtration, followed by drying overnight, the samples were placed in a tube furnace, heated to 800 °Cat 5 °C/min under an atmosphere of H_2/Ar (10%), and kept warm for 2 h. The content of Ca and C in the product was reduced in the above manner. It was proved that after the above treatment, the carbon content reached 99.83%. As shown in Figure S2.

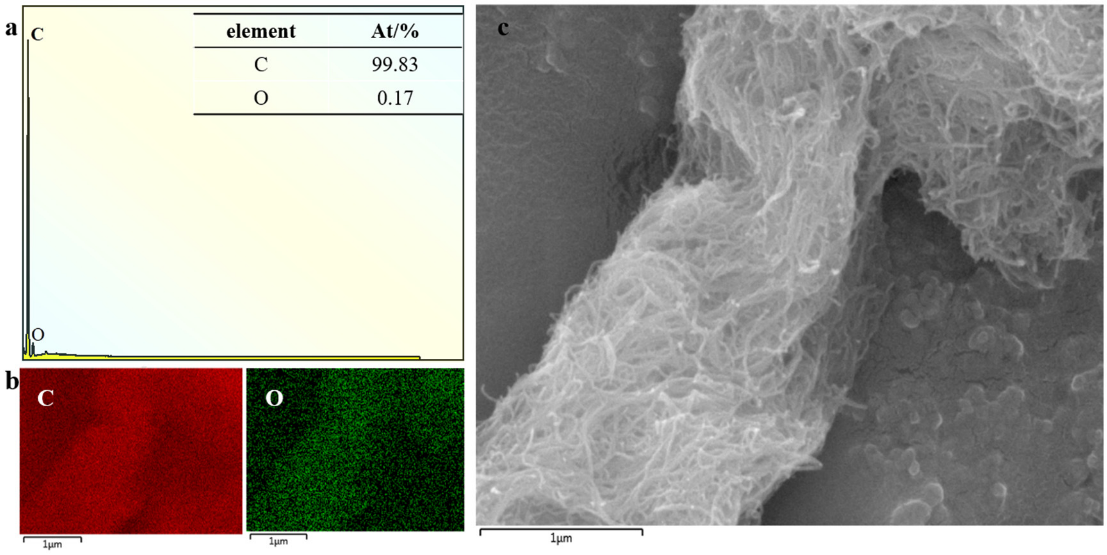

Figure S2. SEM and EDS pictures of purified products, (a) The element content of the product after purification; (b) The EDS map of the purified product, with carbon on the left and oxygen on the right; (c) SEM image of the purified product.
